# Preventing Myocardial Injury Following Non-Cardiac Surgery: A Potential Role for Preoperative Antioxidant Therapy with Ubiquinone

**DOI:** 10.3390/antiox10020276

**Published:** 2021-02-10

**Authors:** Qun Chen, Steven Qi, Laura Hocum-Stone, Edward Lesnefsky, Rosemary F. Kelly, Edward O. McFalls

**Affiliations:** 1 Virginia Commonwealth University Medical Center, Richmond, VA 23219, USA; qun.chen@vcuhealth.org (Q.C.); edward.lesnefsky@vcuhealth.org (E.L.); 2 McGuire Veterans Affairs Medical Center, Richmond, VA 23249, USA; 3 Cardiology-Cardiac Surgery, University of Minnesota, Minneapolis, MN 55455, USA; stevenqi@umn.edu (S.Q.); stone337@umn.edu (L.H.-S.); kelly071@umn.edu (R.F.K.); 4 Minneapolis Veterans Affairs Medical Center, Minneapolis, MN 55417, USA

**Keywords:** CoQ_10_, ubiquinone, myocardial injury, troponin, BNP, vascular surgery, outcomes

## Abstract

Over 240 million non-cardiac operations occur each year and are associated with a 15–20% incidence of adverse perioperative cardiovascular events. Unfortunately, preoperative therapies that have been useful for chronic ischemic heart diseases, such as coronary artery revascularization, antiplatelet agents, and beta-blockers have failed to improve outcomes. In a pre-clinical swine model of ischemic heart disease, we showed that daily administration of ubiquinone (coenzyme Q_10_, CoQ_10_) enhances the antioxidant status of mitochondria within chronically ischemic heart tissue, potentially via a PGC1α-dependent mechanism. In a randomized controlled trial, among high-risk patients undergoing elective vascular surgery, we showed that NT Pro-BNP levels are an important means of risk-stratification during the perioperative period and can be lowered with administration of CoQ_10_ (400 mg/day) for 3 days prior to surgery. The review provides background information for the role of oxidant stress and inflammation during high-risk operations and the potential novel application of ubiquinone as a preoperative antioxidant therapy that might reduce perioperative adverse cardiovascular outcomes.

## 1. Introduction

It has been estimated that over 240 million major non-cardiac surgical procedures are scheduled each year, with at least 1 in 6 suffering from adverse events that occur during those surgeries including a 30-day risk of either death or significant cardiovascular complication [1,2]. Based on the magnitude of these clinical consequences, numerous studies have been completed to focus on strategies that might modify cardiac risks before scheduling major operations that do not involve the heart [3]. In a randomized controlled study involving over 500 veterans undergoing elective vascular surgery and 20 medical centers within the Veterans Affair (VA) health care system, we demonstrated that a strategy of preoperative coronary artery revascularization before an elective vascular surgery did not reduce the long-term risk of death at 2.7 years following the operation [4]. Using an additional preoperative strategy with a preconditioning protocol, we also failed to reduce the incidence of postoperative elevations in cardiac troponin [5]. Other randomized controlled trials have tried to reduce adverse perioperative outcomes and have targeted well-accepted risk factors among patients undergoing elective surgery, including pharmacological agents that are known to reduce secondary outcome measures in patients with coronary artery disease. Unfortunately, they have not consistently shown that the incidence of postoperative adverse cardiovascular events can be mitigated [6,7,8]. Clearly, strategies that modify traditional risks of patients with chronic ischemic heart disease have not proven effective in reducing perioperative ischemic events and myocardial injury (MINS) following high-risk surgery. In a sub-study of our trial, termed the coronary artery revascularization prophylaxis (CARP) trial, we showed that the incidence of elevated cardiac troponin following surgery was not reduced with preoperative coronary artery revascularization, yet was a powerful predictor of long-term risk of adverse outcomes [9]. These data are consistent with other trials [10,11] and emphasize the important fact that cardiac troponins provide incremental value in post-operative risk-stratification [12]. There may be value in assessing cardiac biomarkers following surgery because among patients with an elevated troponin who had their medical regimen maximized, rehospitalization rates following discharge were lower than those individuals with an elevated troponin who did not have a change in therapy [13].

An emerging strategy that has been advocated is the use of preoperative cardiac biomarkers as a means of identifying those individuals at risk for postoperative adverse cardiac events [14]. In that regard, measurement of the cardiac biomarker, Brain Natriuretic Peptide (BNP) before non-cardiac operations is considered a Class I indication, as recommended by the Canadian Cardiovascular Society for Perioperative Care [15]. That guideline was based on evidence that BNP levels correlate with postoperative troponin levels and predict risk of complications within 30 days following surgery [16,17,18]. Of interest, in a randomized controlled trial involving patients with heart failure, these biomarkers were lowered by administration of coenzyme Q_10_ (CoQ_10_) [19]. Conceivably, pharmacological interventions that lower BNP might prove effective in reducing postoperative myocardial injury. In the present review, the goal is to review relevant work from perioperative care and outline how a new approach to reducing oxidant stress and inflammatory signals might reduce injury. The focus of our review is to gather relevant references in perioperative medicine as well as on the potential value in the application of ubiquinone by using a search system that encompasses basic and translational sciences, as defined by approaches recommended and outlined by the PRISMA guidelines.

## 2. Preoperative Risk Assessment and Postoperative Adverse Outcomes

**Figure 1:**
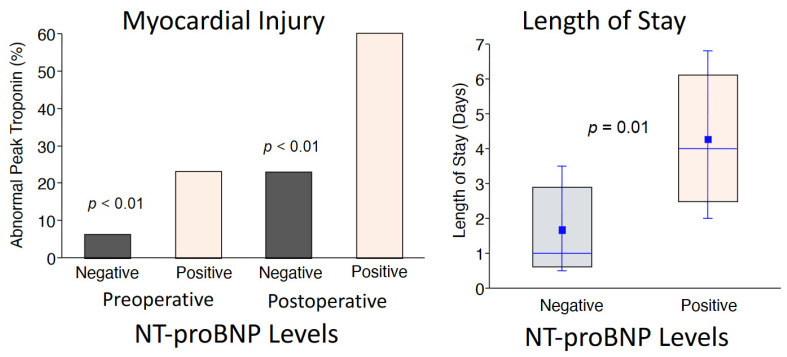
Among the entire group of patients undergoing elective vascular surgery (*n* = 123), an elevated N-terminal pro hormone BNP (NT-proBNP) level predicted those patients who would have myocardial injury (elevated postoperative troponin level) as well as those patients who had a longer postoperative stay in the hospital. These data show the importance of NT-Pro BNP levels before and after the operation for perioperative risk-stratification [2]. Values are expressed as medians, interquartile values, and means. Permission was granted to reproduce the data.

**Figure 2:**
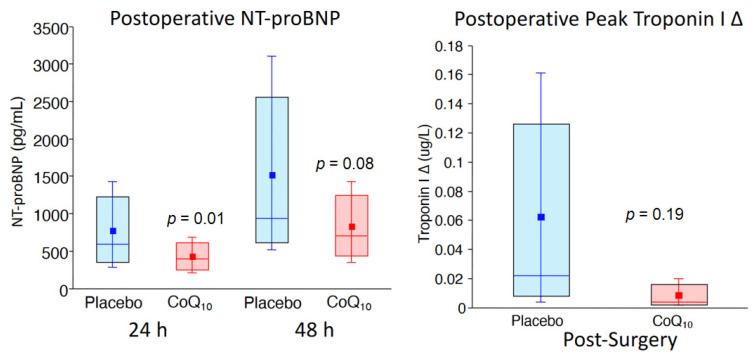
Patients were randomized to either coenzyme Q_10_ (CoQ_10)_ (*n* = 62) or placebo (*n* = 61) for 3 days before elective vascular surgery, and as shown, NT-Pro BNP levels were lower with treatment. The degree of injury, as defined by the change (delta) in troponin I levels from baseline to peak postoperative level, was higher in the placebo compared with that of the CoQ_10_ treatment group [2]. Values are expressed as medians, interquartile values, and means. Permission was granted to reproduce the data.

For over four decades, clinical researchers have developed several models to identify high-risk patients prior to elective vascular surgery [3]. In a comprehensive approach, Lee and colleagues validated six clinical risk variables that independently predict adverse postoperative events following surgery [20]. Termed the Revised Cardiac Risk Index, these variables include a history of coronary artery disease, stroke, heart failure, insulin-dependent diabetes, creatinine > 2.0 mg%, and high-risk operations that include vascular surgery. In a cohort of the CARP trial, we showed that these variables were highly predictive of either death or myocardial infarction within the first 30 days post-surgery, but the risk of adverse events in those subsets could not be lowered with preoperative coronary artery revascularization [21]. Additional randomized controlled studies that targeted therapies for chronic ischemic heart disease, including beta-blockers, aspirin, clonidine, and statins did not show consistent results in preventing myocardial injury following non-cardiac surgery (MINS) [6,7,8,22,23]. In a large cohort of patients undergoing surgery [24], BNP was used as a preoperative biomarker for improving risk-stratification [14] and has now been incorporated into Canadian guidelines for the risk-assessment of patients undergoing elective vascular surgery [25]. Considering that BNP level is a modifiable variable, we completed a pilot study to determine whether preoperative administration of CoQ_10_ might reduce BNP in patients undergoing high-risk vascular surgery. In that regard, the primary results of our randomized controlled trial was that N-terminal pro hormone BNP (NT-proBNP) levels before and after the procedure predicted risk for having myocardial injury as well as having a prolonged post-operative hospitalization period following the elective operation (Figure 1) [2]. These observations are consistent with those of Canadian investigators in use of perioperative cardiac biomarkers prior to non-cardiac operations [25]. Of interest and important for the development of new approaches in perioperative care, we also showed that preoperative administration of coenzyme Q_10_ (CoQ_10_) for 3 days prior to the elective vascular surgical procedure reduced perioperative NT-proBNP levels [2] (Figure 2). These data support the notion that perioperative use of antioxidant therapies may play a critical role in reducing the incidence of cardiac biomarker elevations and possibly improve outcomes following hospital discharge. Clearly, a novel approach to preventing myocardial injury following high-risk surgery is needed. This would require a shift in paradigm, addressing the biological significance of oxidant stress and inflammatory signaling that is incorporated in the pathophysiology of cardiac biomarker release.

## 3. Oxidant Stress and Cardiovascular Disease

Oxidative stress is a well-known factor involved in cardiovascular diseases including ischemia-reperfusion and heart failure [26]. While a small amount of ROS (reactive oxygen species) may play a physiological role in signaling transduction, uncontrolled ROS formation, especially during early reperfusion, increases oxidative stress and cell injury [26,27,28]. The burst in ROS generation is a critical factor in the increased opening of mitochondrial permeability transition pores (MPTPs) that increases cell injury during ischemia-reperfusion [29,30]. The MPTP is a non-selective pore that is located in inner mitochondrial membranes [31]. MPTP opening leads to increased permeability of the inner membrane that triggers mitochondrial damage. A permeabilized inner membrane during MPTP opening may augment ROS generation by increasing electron flow into the electron transport chain due to uncoupled respiration [32]. The permeation of the inner membrane leads to decreased ATP generation by depolarizing inner mitochondria membrane potential [31]. MPTP opening also leads to increased permeability of the outer membrane by inducing mitochondrial matrix swelling through accumulation of calcium and H_2_O within the matrix [33]. Proteins located within the mitochondrial intermembrane space, including cytochrome *c* and apoptosis-inducing factor (AIF), are translocated into the cytosol through the leaky outer membrane and increase apoptosis in both a caspase-dependent and caspase-independent manner [34,35,36]. An increase in ROS generation also impairs cardiac function by oxidizing the proteins involved in cardiac contractile activity [37]. ROS generation impairs mitochondrial quality control mechanisms including inhibition of autophagy, which is a key process for removing dysfunctional mitochondria in the heart [38]. The increased ROS production also increases apoptotic cell death and cardiac hypertrophy by activating signaling transduction [39]. ROS generation also increases ventricular fibrosis, including in aged hearts, by activating cardiac fibroblasts [39,40]. ROS generation can also increase ER (endoplasmic reticulum) stress [41,42] that contributes to mitochondrial dysfunction and cardiac injury during aging [43]. The ER not only plays a critical role in protein folding and lipid synthesis but is a calcium storage site and regulates calcium homeostasis [41,43]. An increase in misfolded proteins within the ER causes ER dysfunction (ER stress) [44]. The initial response to the ER stress is an attempt to restore ER function by slowing down protein synthesis. However, prolonged ER stress increases cell injury. Induction of acute ER stress using thapsigargin increases cell death by impairing mitochondrial function in adult hearts [44,45,46,47]. ER stress is increased in the heart with aging [43]. Chronic treatment with 4-phenylbutyrate (4-PBA), which is a chemical chaperone that stabilizes protein conformation within the ER, improves mitochondrial function in aged hearts [43], supporting the notion that ER stress contributes to mitochondrial dysfunction during aging. Although the mechanisms by which aging leads to increased ER stress remains unclear, ROS generation is a potential causative factor. Therefore, future studies should consider whether the attenuation of ROS generation by overexpression of either catalase [48] or CoQ treatment [19] can decrease ER stress in aged hearts.

In addition to the direct damage caused by free radicals, oxidative stress can increase mitochondrial and cell injury by facilitating proteases, including calpain activation [49]. Calpains are a family of calcium-activated cysteine proteases that include 14 isoforms [49]. Ubiquitous calpains, including calpain 1 and calpain 2, exist in cytosol and mitochondria [49,50], and their activation increases tissue injury during myocardial ischemia and reperfusion [34,51,52,53,54,55,56,57]. In fact, the activities of calpain 1 and calpain 2 are both increased in isolated hearts following ischemia-reperfusion [49]. Cytosolic calpain 1 activation leads to cleavage of proteins including bid, Na^+^,K^+^-ATPase, Ca^2+^-ATPase, spectrin, and troponin T [49,55,56,57]. Activation of calpain 1 impairs cardiac function by degrading contractile proteins including junctophilin-2 [58]. Ischemia-reperfusion also leads to increased activities of mitochondrial calpain 1 [34] and calpain 2 [50]. Activation of mitochondrial calpain 1 and calpain 2 leads to a damaged electron transport chain (ETC) and MPTPs that are sensitized to opening [34,50,54,59]. Interestingly, elevated calcium concentrations, even in pathological conditions, are below the threshold to activate calpain 1 and calpain 2 [49]. However, the calcium concentration required to activate calpain 1 and calpain 2 is markedly decreased in the presence of oxidative stress [60,61]. Thus, increased ROS generation may be a key co-factor to activating calpain 1 and calpain 2 during cardiac stress states including catecholamine exposure, ischemia-reperfusion, heart failure, and aging [62]. Thus, ROS generation at the time of a major, high-risk operation can lead to cell injury through direct oxidation of proteins and indirectly facilitate proteases activation.

In cardiac myocytes, most ROS are generated from the electron transport chain (ETC) [26,27,28]. Superoxide anion (O_2_^•−^) is the most abundant ROS in cells [63]. The superoxide anion is formed when O_2_ captures an additional electron leaking from the ETC. Superoxide anions are the base for generating other types of ROS, including hydrogen peroxide (H_2_O_2_), hydroxyl (OH^•^), and peroxynitrite (ONOO^−^). Since most ROS are generated at the ETC, it is not surprising that the initial target of ROS damage is in the ETC itself [64]. Prevention of electron flow into the ETC leads to decreased ROS generation in control [65] and ischemia-damaged mitochondria [66,67]. Inhibition of proximal electron flow through the ETC reduces cardiac injury during reperfusion by decreasing ROS generation [30,68,69] and calcium overload [69]. Blockade of the electron transport also decreases cardiac injury in aged hearts following ischemia-reperfusion [70,71]. These results indicate that the mitochondrial respiratory chain is a key source of ROS production in cardiac myocytes. ROS generated by the ETC first impairs the ETC itself to further augment ROS generation [66]. Thus, the damaged ETC plays a central role in ROS generation [63] and, in that regard, may be an important pharmacological target during major operations.

Complexes I, II, and III are potential sites for ROS production by the ETC [26,72,73]. Complex I is the first respiratory complex and consists of a membrane arm embedded in the inner membrane and a peripheral arm oriented into the mitochondrial matrix [74,75]. The peripheral arm is responsible for NADH oxidation and subsequent electron transfer through complex I to ubiquinone [74]. The membrane arm is essential for proton pumping across the inner membrane. Although complex I is a key site of ROS generation, the exact sites (subunits) of ROS generation within complex I are poorly defined. Subunits in both the membrane arm, including N2, and the peripheral arm, including flavin mononucleotide (FMN), are proposed to be sites of ROS generation within complex I [76,77]. Complex I generates ROS through two mechanisms: forward (complex I → Q → complex III) or reverse (complex II → Q → complex I) electron flow-mediated ROS generation [78,79]. The forward flow-induced ROS generation requires an almost fully reduced condition within complex I [80]. This situation usually occurs when complex I is severely damaged or in the presence of complex I inhibitors including rotenone [65,68,81]. Ischemia-reperfusion damages complex I at its quinone binding sites, which leads to electron accumulation within complex I and increases forward flow-mediated ROS generation [82]. Inhibition of complex I using rotenone also increases ROS generation from complex I [65]. Blockade of electron transport at the distal site of the ETC including cytochrome oxidase also favors forward flow-induced ROS generation from complex I [62,83].

In addition to blocking electron transport at the individual respiratory complexes, disruption of supercomplexes can contribute to decreased oxidative phosphorylation and increased ROS generation [84]. The supercomplexes are assembled with complexes I, III, and IV in the ratio of 1:2:1 [62]. Formation of the supercomplexes increases the efficiency of electron transport and decreases ROS generation by reducing electron leakage from the ETC [85,86]. Destabilization of supercomplexes increases ROS generation from complex I [87,88]. The content of supercomplexes is decreased in mitochondria from aged or failing hearts [62]. This may lead to increased ROS generation from complex I during aging and heart failure.

Complex I can also produce ROS by inducing the reverse electron flow that occurs when succinate is used as a complex II substrate to provide electron flow from complex II to complex I [79,89]. Mitochondrial membrane potential is a driving force for the electron flow from complex II to complex I [78]. Thus, depolarization of mitochondrial membrane potential using an uncoupler [78,79] is an efficient approach to eliminate the reverse flow-induced ROS generation. In addition, blockage of electron transport from complex II to I by using complex I [90] or complex II inhibitors also decreases the ROS generation by the reverse electron flow [79,89]. ROS generated by reverse electron flow also increases cell injury during ischemia-reperfusion [91,92].

As discussed above, complex II plays a role in reverse flow-induced ROS generation. The complex II may also directly generate ROS within complex II [93,94]. ROS generation by complex II is dependent on the succinate concentration [79]. High concentration of succinate (>5 mM used in most in vitro analysis) inhibits ROS generation from complex II. ROS generation is increased in complex II when a relatively low concentration of succinate (0.5 mM) is used in the presence of complex II inhibitor (thenoyltrifluoroacetone (TTFA)) or a complex III Qo center inhibitor [93]. TTFA blocks electron transport at the terminal part of complex II, which increases electron accumulation within complex II and subsequent ROS generation [93]. In contrast, inhibition of complex II at the succinate-binding site with malonate decreases ROS generation from complex II due to decreased electron flowing into complex II [65].

Complex III is a key source of ROS generation in heart mitochondria [65,72]. ROS is generated in both the complex III Qo center (quinol oxidation site oriented to the mitochondrial intermembrane space) and the Qi center oriented to the mitochondrial matrix space. ROS produced at the Qo center are oriented to the intermembrane space and subsequent cytoplasm through voltage-dependent anion channel (VDAC) in the outer mitochondrial membrane [65,72,95,96]. ROS generated at the Qi center are released into the mitochondrial matrix and detoxified by mitochondrial antioxidants. Antimycin A is a classic complex III inhibitor that inhibits electron transport at the Qi center [65]. Antimycin A predominantly increases ROS generation from the Qo center. Myxothiazol or stigmatellin inhibit electron transport at the complex III Qo center [65,97]. Thus, inhibition of complex III using myxothiazol or stigmatellin leads to decreased ROS generation from complex III. As discussed above, myxothiazol or stigmatellin increases ROS generation from complex I or complex II based on the substrate usage. ROS generated from the complex III Qo center plays a critical role in aging and ischemia-reperfusion injury [97]. Recent study also showed that ROS generated by the Qo site of complex III increases ER (endoplasmic reticulum) stress [42].

The electron transport chain is not the only source of oxidant stress within mitochondria of heart tissue [27,62]. Monoamine oxidase (MAO), p66^shc^, and NOx4 are potential sources of ROS generation within mitochondria. MAO is located on the outer mitochondrial membrane and functions in the regulation of catecholamines and other biogenic amines [37]. MAO has two isoforms: MAO-A and MAO-B. Both MAO-A and B isoforms are equally expressed in human hearts. However, MAO-A is the major isoform in rat hearts, and the MAO-B is the major isoform in mouse hearts [37]. H_2_O_2_ is generated when MAO breaks down neurotransmitters, including norepinephrine, epinephrine, and dopamine [98]. MAO-mediated ROS generation contributes to cardiac injury [99], muscle dystrophy [100], and aging.

Another redox enzyme that exists in the mitochondrial intermembrane space is p66^shc^. It is one of the isoforms in the ShcA adaptor protein family [62,101 and has a cytochrome *c* binding region towards the N-terminal side end [101]. The cytochrome *c* binding domain is the redox center of p66^Shc^ [101,102]. In conditions with increased ROS generation, including cardiac ischemia, the cytochrome *c* binding domain in p66^Shc^ binds with cytochrome *c* leading to electrons transferring from cytochrome *c* to oxygen to increase ROS generation [101]. Downregulation of p66^Shc^ leads to decreased ROS generation and prolonged mouse life span, indicating that ROS generation from p66^Shc^ contributes to the aging process [103,104].

## 4. Antioxidants and Cardioprotection

Cells have antioxidant defenses to detoxify the ROS [63]. The antioxidant enzymes include superoxide dismutase (SOD), catalase, and glutathione peroxidase. SODs are the most effective antioxidant enzymes at converting superoxide anion (O_2_^●−^) to hydrogen peroxide (H_2_O_2_). SODs also inhibit the formation of peroxynitrite (ONOO^−^) by preventing NO reaction with superoxides via the timely removal of superoxides [105]. SOD has three isoforms: cytosolic SOD1, mitochondrial SOD2, and extracellular SOD3. Since copper (Cu) and zinc (Zn) are required as cofactors for SOD1 and SOD3, these SODs are also called Cu-ZnSOD. SOD2 is also referred to as Mn(manganese)SOD in that the Mn is used as a cofactor in SOD2 [105]. Stimulation of SOD1/2 expression decreases cerebral injury during heart arrest and resuscitation [106]. Genetic disruption of the SOD2 gene increases oxidative stress and cardiac hypertrophy [107], supporting the concept that superoxide anions generated from mitochondria play a critical role in oxidative stress-induced cell injury.

Hydrogen peroxide is detoxified by antioxidants including catalase, glutathione peroxidase (GPX), and peroxiredoxins (PRDX) [63]. Catalase reduces H_2_O_2_ to H_2_O, especially in the presence of high concentrations of hydrogen peroxide, and also functions as a peroxidase in conditions with low concentrations of hydrogen peroxide [108,109]. Aging leads to myocardial hypertrophy and dilatation, cardiac dysfunction, and increased fibrosis. Overexpression of mitochondrial-targeted catalase reverses these defects in aged mice. These results clearly show that increased mitochondrial oxidative stress contributes to cardiac dysfunction during aging [48].

Peroxiredoxins (PRDXs) reduce hydrogen peroxide and peroxynitrite [110]. PRDXs are a family of thiol specific antioxidant proteins including six isoforms in mammalian cells. PRDX 1, 2, 3, and 6 mainly exist in the cytosol. PRDX 4 is found in the endoplasmic reticulum [111]. In addition, PRDX 3 and 5 are present in the mitochondria [90,111]. Ischemia-reperfusion leads to decreased PRDX 3 activity in mouse heart mitochondria [112]. Overexpression of the PRDX3 decreases the development of myocardial infarction-induced heart failure [113]. Genetic inhibition of the PRDX 6 increases cell injury during ischemia-reperfusion by increasing lipid peroxidation [114]. In contrast, overexpression of PRDX 6 decreases cardiac injury during oxidative stress [115]. Interestingly, knockout of p53 leads to increased PRDX3 content in mouse hearts in the basal condition. Knockout of p53 improves mitochondrial function and decreased cardiac injury during ischemia-reperfusion [90], supporting that PRDXs play a critical role in reducing ROS generation from mitochondria during ischemia-reperfusion.

Glutathione peroxidases (Gpx), with six isoforms, are located in the cytoplasm, nuclei, and mitochondria [116]. Glutathione (GSH) is the major intracellular thiol reserve, mainly present in the reduced form. GSSG is glutathione disulfide. The Gpx reduces H_2_O_2_ to H_2_O and simultaneously oxidizes GSH to GSSG. Then, the GSSG is reduced to GSH by glutathione reductase in the presence of NAPDH. The GSH/GSSG ratio is used as a marker of oxidative stress. Ischemia-reperfusion leads to decreased Gpx4 expression [117]. Resveratrol treatment protects cells exposed to exogenous H_2_O_2_ by increasing glutathione peroxidase, catalase, and heme oxygenase-1 (HO-1), indicating that resveratrol treatment increases vascular oxidative stress resistance by scavenging H_2_O_2_ [118]. These results clearly show that glutathione peroxidase plays a critical in deceasing oxidative stress during pathological conditions.

Heme oxygenase (HO) catabolizes heme to produce labile Fe, carbon monoxide (CO), and biliverdin [119]. Free heme increases the production of hydroxyl radicals through the Fenton reaction (4). HOs include two isoforms: HO-1 and HO-2. HO-1 is an inducible form. Heme catabolism by HO-1 extracts Fe from the protoporphyrin IX ring to produce labile Fe that is buried in a multimeric complex to prevent labile Fe from triggering the Fenton reaction [119]. Heme catabolism also produces biliverdin that is converted to bilirubin, having an antioxidant effect. CO (carbon monoxide) is also produced during heme catabolism [119]. Although an increasing amount of evidence indicates that oxidative stress plays a key role in cell damage during heart failure [120], ischemia-reperfusion, and aging, the effect of administering exogenous antioxidant treatment and reducing cell injury during pathological conditions including aging and heart failure is still controversial [121]. As discussed above, overexpression of catalase improves cardiac function in aged hearts [48], indicating that promotion of endogenous antioxidants may be a proper strategy to decrease oxidative stress. Interestingly, administration of dimethyl fumarate leads to increased Nrf2 and HO-1 expression [122]. Dimethyl fumarate treatment also decreases cell injury during ischemia-reperfusion by increasing HO-1 expression [123,124]. Stimulation of HO-1 expression with dimethyl fumarate treatment may be a novel approach to decrease cell injury during aging because endogenous antioxidants are already impaired [121].

## 5. Potential Mechanisms of CoQ_10_ Against Oxidant Stress

**Figure 3:**
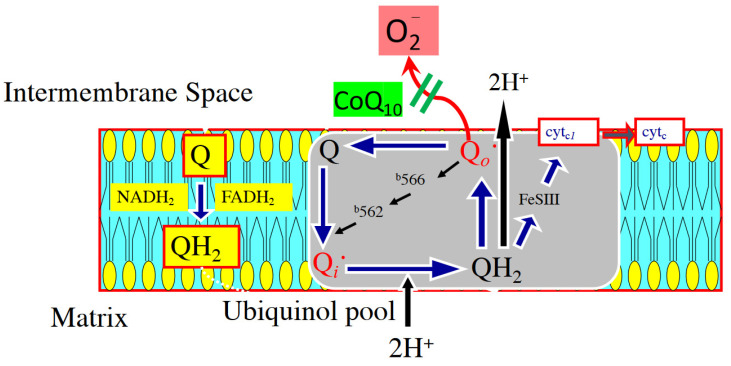
The figure demonstrates a potential mechanism for the addition of CoQ_10_ in the diet for 4 weeks and how enhanced electron transport chain functioning through complex III (Q-cycle) reduces the generation of reactive oxygen species in the intermembrane space [128].

Coenzyme Q_10_ (CoQ_10_), or ubiquinone, is a lipid-soluble benzoquinone with 10 isoprenyl units in its side chain. It plays a key role as an intracellular antioxidant, protecting membrane phospholipids and mitochondrial membrane proteins from free radical-induced oxidative injury [2]. Functioning within the inner mitochondrial membrane [2], ubiquinone serves as a structural component of complexes I and III and facilitates the transport of electrons to their ultimate reaction with oxygen for water production. In this capacity, the synthesis of adenosine triphosphate within the electron transport chain of myocytes is critical [125,126]. CoQ_10_ also prevents the leakage of electrons to oxygen that would result in the production and release of reactive oxygen species (ROS) (Figure 3).

In addition to its actions on electron transport, CoQ_10_ binds to common sites involved with the MPTP, preventing pore formation and membrane depolarization, both of which trigger apoptosis. The mechanism by which CoQ_10_ inhibits pore formation in mitochondria involves secondary changes in MPTPs’ calcium binding affinity, preventing cytochrome *c* release and subsequent ATP hydrolysis [127]. The protection of complex I activity during ischemia-reperfusion by inhibiting calpain 1 and 2 leads to a reduction in ROS generation [75]. Additional experimental work from various animal models as well as patients supports the notion that supplementation of CoQ_10_ has value in reducing oxidant stress [128]. In a rat model of Alzheimer’s disease, cultured cortical neuron induced-damage by exposure to amyloid-beta can be inhibited with the addition of CoQ_10_ and ROS can be reduced through a mechanism involving activation of the PI3-K/Akt survival pathway [129]. In a swine model, dietary supplementation of CoQ_10_ (5 mg/kg/day) for 30 days increased the myocardial content of ubiquinone in isolated mitochondria by 30%. When the pig hearts were then placed on cardiopulmonary bypass and subjected to 30 min of regional ischemia-reperfusion, CoQ_10_ treated hearts showed improved left ventricular (LV) function, lower levels of creatine kinase release, and reduced levels of malonaldehyde (MDA) content, a marker of oxidant stress within post-ischemic tissue [130].

**Figure 4:**
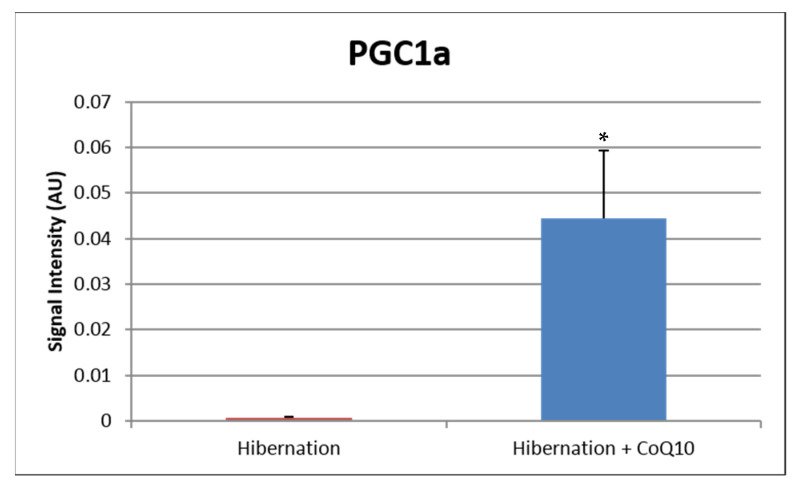
In a pre-clinical swine study of chronic myocardial ischemia, administration of CoQ_10_ (400 mg/day) in the chow for 4 weeks increased the expression of activated PGC1-alpha, the master switch for mitochondrial biogenesis, leading to enhanced expression of mitochondrial antioxidant proteins in the chronically ischemic territory. The data support the concept that CoQ_10_ is protective by transcriptionally regulating antioxidant proteins in heart tissue. * *p* < 0.05.

Similar observations have been shown in patients pretreated with CoQ_10_ prior to coronary artery bypass graft surgery (CABG) [2], with reduced levels of MDA and protein carbonyls and enhanced glutathione peroxidase activity observed post-CABG [131,132]. Although CoQ_10_ levels may have a direct effect on reducing oxidant stress, chronic administration may also improve the antioxidant status indirectly at the transcriptional level by the regulation of mitochondrial protein expression. In skeletal muscle tissue, aging reduces the levels of peroxisome proliferator-activated receptor gamma coactivator 1-alpha (PGC-1α), the master switch of mitochondrial biogenesis. This reduced PGC-1α level leads to depleted concentrations of glutathione (GSH) with enhanced oxidative stress markers [133]. In studies of isolated C2C12 skeletal muscle cells [128], supplementation of CoQ_10_ with α-lipoic acid enhanced PGC-1α expression and increased genes that encode proteins involved in glutathione synthesis, recycling, and metabolism [134]. These findings are consistent with observations made in a rat model of pharmacologically-induced seizures, whereby administration of CoQ_10_ reduced oxidant stress by enhancing PGC-1α nearly 3-fold [135]. These changes were also associated with increased levels of nuclear factor erythroid 2-related factor 2 (Nrf2) and silencing information regulator 1 (Sirt1), both of which improve redox control within the cell, by increasing mitochondrial antioxidants such as superoxide dismutase 2. It has been suggested that CoQ_10_ increases the expression and activity of PGC-1α by its activation of the cAMP response element binding protein and adenosine monophosphate-activated protein kinase (AMPK) phosphorylation [136]. In our swine model of chronic myocardial ischemia, we administered daily CoQ_10_ (400 mg/day) for 4 weeks, and compared to placebo-treated animals, we observed an enhanced expression of nuclear-bound PGC1-alpha, indicating activation of mitochondrial biogenesis, as well as increased expression of antioxidant proteins within isolated mitochondria [137] (Figure 4 and Table 1). In addition to its role in mitochondrial biogenesis and protection, CoQ_10_, the only endogenously produced lipid-soluble antioxidant in the cell, is also present within the Golgi apparatus, and plays a key role in redox control and nitric oxide elaboration by maintaining coupling and normal activity of eNOS [138]. Taken together, these data support the concept that CoQ_10_ provides a key role as an antioxidant in the heart cell by enhancing ETC exchange within mitochondria as well as increasing expression of antioxidant proteins to reduce the accumulation of oxidant stress within cardiac tissue [128].

**Figure 5:**
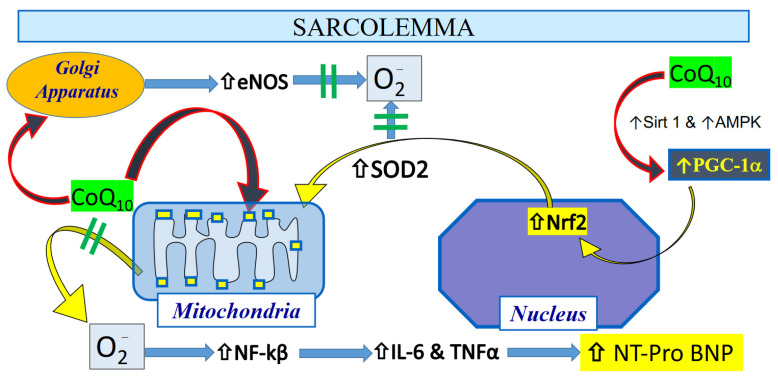
The possible mechanism of protection of the heart following administration of CoQ_10_ relates to a direct antioxidant effect as well as an indirect effect on mitigating inflammatory pathways that can lead to increased NT-Pro BNP levels in stressed heart tissue [2]. Permission was granted to reproduce the schema.

Reducing oxidant stress in the mitochondria within the myocyte may play a key role in attenuating inflammation within cardiac tissue. In a meta-analysis, CoQ_10_ administration was shown to significantly decrease plasma C-reactive protein (CRP) levels [139]. A number of cytokines, including interleukin-6 (IL-6) [2], are secondary messengers that activate production and release of CRP in the liver [140] and are reduced following administration of CoQ_10_ [135]. The greatest effect on CRP reduction with CoQ_10_ administration is observed among those patients with increased IL-6 levels, providing support for the notion that the hepatic release of CRP is downstream from IL-6 [141]. A reasonable mechanism combining CoQ_10′_s antioxidant effect with mitigation of inflammation is by the inhibition of NF-kB, which transcriptionally regulates the production and elaboration of pro-inflammatory cytokines [142]. Very importantly, this regulation of inflammatory cytokine markers is activated by complex I-generated ROS [143,144], providing additional support for the importance of the Q-cycle within the inner mitochondrial membrane of hearts as a key regulator of inflammation (Figure 5).

## 6. Administration of CoQ_10_ and Improved Clinical Outcomes

Among older individuals, the concentration of plasma CoQ_10_ levels is low, but when increased, inversely correlates with levels of lipid oxidative biproducts [145]. CoQ_10_ supplementation is well tolerated with doses of at least 2400 mg/day [146] and is protective against oxidative stress with a number of cardiovascular and neurodegenerative diseases [147,148]. In patients scheduled for open heart surgery, CoQ_10_ administration lowers the requirement for inotropic drugs following surgery, with an observed reduction in the number of arrhythmias [149]. In elderly patients from Sweden, giving CoQ_10_ (200 mg/day) with selenium (200 µg as selenized yeast) was associated with reduced cardiovascular deaths after 4 years, an observation that was also observed 10 years post-randomization [150]. In addition to the improved outcomes, CoQ_10_ supplementation reduced elevated cardiac biomarkers in the blood that have been used as identifiers of poor outcomes. In a group of dialysis patients who were randomized to receive daily CoQ_10_ (1200 mg/day), plasma concentrations of oxidant stress markers, F2-isoprostanes, were lower after 4 months compared with that of placebo-treated patients [151]. Very interestingly, among a prespecified group of individuals, therapy reduced troponin-T and NT pro-BNP levels, providing additional evidence for a critical link between oxidant stress and these commonly used cardiac biomarkers. This observation is important for interpreting the results of the Q-SYMBIO trial, which among patients with stable congestive heart failure tested the benefit of chronic administration of CoQ_10_ (300 mg/day) versus placebo. The design of the trial was a double-blind, randomized, controlled study and showed that treatment led to a significant long-term reduction in major cardiovascular endpoints [19]. Consistent with other studies, treatment also lowered plasma BNP levels, which is an important cardiac biomarker for predicting adverse outcomes in patients with heart failure. Among healthy elderly patients, serum levels of ubiquinol are correlated with reduced levels of NT pro-BNP [152], and when treatment is provided, it further lowers BNP levels over a period of 5 years [150,153]. The mechanism by which CoQ_10_ reduces BNP is unclear but may be related to its effects on reducing either oxidant stress or inflammation. In a cohort of 51 patients with stable ischemic heart disease, those patients who were randomized to CoQ_10_ (300 mg/day) for 4 months had increased activity of antioxidant enzymes, such as superoxide dismutase, catalase, and glutathione peroxidase, and reduced inflammatory markers TNF-α and IL-6 [154]. The effects of CoQ_10_ administration on reducing the inflammatory cytokines in heart tissue is critical for the interpretation of the Q-SYMBIO trial results in patients with congestive heart failure [2]. Elaboration of the cytokine IL-6 occurs within cardiac myocytes and with sustained activation of gp130, a member of the signaling pathway, and induces adverse remodeling in the heart, such as hypertrophy. At 6 weeks following transaortic constriction (TAC) in wild type mice, the degree of hypertrophy was activated by a combination of CaMKII and the STAT3 pathways, and this signaling cascade in addition to the ventricular hypertrophy was blocked in those mice with disruption of the IL-6 gene [155]. The degree of BNP expression was also lowered in the IL-6 knock-out mice following TAC, providing additional evidence that the elaboration of BNP in the heart is downstream from cytokine activation of IL-6. Furthermore, among those patients with insulin resistance and rheumatoid arthritis, increased IL-6 levels were the best predictor of increased NT-proBNP levels, further supporting a direct link between inflammation and elevated cardiac biomarkers [156]. These clinical observations are also supported by work in human cardiac fibroblasts, which has shown increased BNP expression following exposure to various inflammatory markers, including the interleukins [2,157].

In summary, traditional therapies that improve outcomes in patients with chronic ischemic heart disease have failed to reduce perioperative adverse outcomes following non-cardiac operations. Use of cardiac biomarkers such as NT-proBNP has become an important means of risk-stratification and can identify those patients who will experience myocardial injury as defined by elevated cardiac troponin following surgery. There is emerging evidence that administration of ubiquinone prior to a major cardiac operation will reduce these elevations in BNP and troponin by mitigating oxidant stress [158]. However, the number of studies that have used rigorous designs involving randomized, controlled, double-blind strategies with treatment of CoQ_10_ versus placebo prior to major vascular or cardiac operations are limited (Table 2). Clearly, additional studies are needed, particularly related to the proper dosing of ubiquinone relative to plasma levels, as well to understanding the potential benefits in patients with congestive heart failure relative to oxidant stress markers and improved bioenergetics [159]. Hopefully, the community dealing with perioperative care can advance novel, alternative, safe antioxidant therapies prior to and following major vascular operations, particularly among high risk patients defined by either the revised cardiac risk index [20] or an elevated preoperative level of BNP [14] as a means of reducing short-term and potentially long-term postoperative adverse outcomes.

## Figures and Tables

**Table 1:** Mitochondrial anti-oxidant proteins.

Antioxidant Protein	Accession #	*p* Value
Glutathione peroxidase	A0A287AG70_PIG	0.029
Superoxide dismutase	A0A287A4Z2_PIG	0.001
Aldehyde dehydrogenase 6	F1S3H1_PIG	0.002
Superoxide dismutase (Cu-Zn)	SODC_PIG	0.87
Glutathione S-transferase kappa	F1SRV4_PIG	0.6
Cluster of aldehyde dehydrogenase	F1SDC7_PIG [4]	0.54
Alcohol dehydrogenase	F1RTZ1_PIG	0.62
Thioredoxin reductase 2	A0A287BQ74_PIG	0.99
Glutathione-disulfide reductase	F1RX66_PIG	0.93

*n* = 4/group; Data normalized to healthy animals; Log fold change calculated by Hib + CoQ_10_/Hibernation. Significance determined by the Permutation test with the Benjamini–Hochberg test; Resulting *p* value: *p* < 0.00834.

**Table 2:** Randomized, controlled, double-blind studies testing the effects of preoperative treatment with CoQ_10_ vs. placebo on postoperative outcome measures.

Study Results of Treatment	Type of Surgery	Sample Size	Pre-Op Rx Time	Dose of CoQ_10_	Primary End Point Measure	Post-Op Time
Khan et al. [2]	Vascular	*n* = 121	3 days	400 mg/d	NT Pro-BNP	30 days
Orlando et al. [160]	AVR	*n* = 50	7 days	400 mg/d	Troponin I/CK-MB	5 days
Rosenfeldt et al. [131]	CABG ± AVR	*n* = 121	14 days	300 mg/d	MDA	30 days
Taggart et al. [161]	CABG	*n* = 20	12 h	600 mg	Troponin T/CKMB	30 days
Judy et al. [162]	CABG ± AVR	*n* = 20	14 days	100 mg/d	CI/LVEF	30 days

MDA (malondialdehyde); CK-MB (creatine kinase-myocardial band); CI (cardiac index), LVEF (left ventricular ejection fraction), CABG (coronary artery bypass graft), AVR (aortic valve replacement).

## References

[1] Weiser T.G., Regenbogen S.E., Thompson K.D., Haynes A.B., Lipsitz S.R., Berry W.R., Gawande A.A. (2008). An estimation of the global volume of surgery: A modelling strategy based on available data. Lancet.

[2] Khan A., Johnson D.K., Carlson S., Hocum-Stone L., Kelly R.F., Gravely A.A., Mbai M., Green D.L., Santilli S., Garcia S. (2020). NT-Pro BNP predicts myocardial injury post-vascular surgery and is reduced with CoQ(10): A randomized double-blind trial. Ann. Vasc. Surg..

[3] Eagle K.A., Berger P.B., Calkins H., Chaitman B.R., Ewy G.A., Fleischmann K.E., Fleisher L.A., Froehlich J.B., Gusberg R.J., Leppo J.A. (2002). ACC/AHA guideline update for perioperative cardiovascular evaluation for noncardiac surgery—Executive summary: A report of the American college of cardiology/American heart association task force on practice guidelines (Committee to update the 1996 guidelines on perioperative cardiovascular evaluation for noncardiac surgery). J. Am. Coll. Cardiol..

[4] McFalls E.O., Ward H.B., Moritz T.E., Goldman S., Krupski W.C., Littooy F., Pierpont G., Santilli S., Rapp J., Hattler B. (2004). Coronary-artery revascularization before elective major vascular surgery. N. Engl. J. Med..

[5] Garcia S., Rector T.S., Zakharova M., Herrmann R.R., Adabag S., Bertog S., Sandoval Y., Santilli S., Brilakis E., McFalls E.O. (2016). Cardiac remote ischemic preconditioning prior to elective vascular surgery (CRIPES): A prospective, randomized, sham-controlled phase II clinical trial. J. Am. Heart Assoc..

[6] Devereaux P.J., Yang H., Yusuf S., Guyatt G., Leslie K., Villar J.C., Xavier D., Chrolavicius S., Greenspan L., Pogue J. (2008). Effects of extended-release metoprolol succinate in patients undergoing non-cardiac surgery (POISE trial): A randomised controlled trial. Lancet.

[7] Biccard B.M., Sigamani A., Chan M.T.V., Sessler D.I., Kurz A., Tittley J.G., Rapanos T., Harlock J., Szalay D., Tiboni M.E. (2018). Effect of aspirin in vascular surgery in patients from a randomized clinical trial (POISE-2). Br. J. Surg..

[8] Berwanger O., de Barros E.S.P.G., Barbosa R.R., Precoma D.B., Figueiredo E.L., Hajjar L.A., Kruel C.D., Alboim C., Almeida A.P., Dracoulakis M.D. (2017). Atorvastatin for high-risk statin-naïve patients undergoing noncardiac surgery: The lowering the risk of operative complications using atorvastatin loading dose (LOAD) randomized trial. Am. Heart J..

[9] McFalls E.O., Ward H.B., Moritz T.E., Apple F.S., Goldman S., Pierpont G., Larsen G.C., Hattler B., Shunk K., Littooy F. (2008). Predictors and outcomes of a perioperative myocardial infarction following elective vascular surgery in patients with documented coronary artery disease: Results of the CARP trial. Eur. Heart J..

[10] Devereaux P.J., Biccard B.M., Sigamani A., Xavier D., Chan M.T.V., Srinathan S.K., Walsh M., Abraham V., Pearse R., Wang C.Y. (2017). Association of postoperative high-sensitivity troponin levels with myocardial injury and 30-day mortality among patients undergoing noncardiac surgery. Jama.

[11] Biccard B.M., Scott D.J.A., Chan M.T.V., Archbold A., Wang C.Y., Sigamani A., Urrútia G., Cruz P., Srinathan S.K., Szalay D. (2018). Myocardial injury after noncardiac surgery (MINS) in vascular surgical patients: A prospective observational cohort study. Ann. Surg..

[12] Marston N., Brenes J., Garcia S., Kuskowski M., Adabag S., Santilli S., McFalls E.O. (2012). Peak postoperative troponin levels outperform preoperative cardiac risk indices as predictors of long-term mortality after vascular surgery Troponins and postoperative outcomes. J. Crit. Care.

[13] Sandoval Y., Zakharova M., Rector T.S., Brilakis E.S., Drexel T., McFalls E.O., Garcia S. (2016). Frequency of increase in cardiac troponin levels after peripheral arterial operations (carotid endarterectomy, abdominal aorta procedure, distal bypass) and their effect on medical management. Am. J. Cardiol..

[14] Rodseth R.N., Biccard B.M., Le Manach Y., Sessler D.I., Lurati Buse G.A., Thabane L., Schutt R.C., Bolliger D., Cagini L., Cardinale D. (2014). The prognostic value of pre-operative and post-operative B-type natriuretic peptides in patients undergoing noncardiac surgery: B-type natriuretic peptide and N-terminal fragment of pro-B-type natriuretic peptide: A systematic review and individual patient data meta-analysis. J. Am. Coll. Cardiol..

[15] Duceppe E., Parlow J., MacDonald P., Lyons K., McMullen M., Srinathan S., Graham M., Tandon V., Styles K., Bessissow A. (2017). Canadian cardiovascular society guidelines on perioperative cardiac risk assessment and management for patients who undergo noncardiac surgery. Can. J. Cardiol..

[16] Kusumoto A., Miyata M., Kubozono T., Ikeda Y., Shinsato T., Kuwahata S., Fujita S., Takasaki K., Yuasa T., Hamasaki S. (2012). Highly sensitive cardiac troponin T in heart failure: Comparison with echocardiographic parameters and natriuretic peptides. J. Cardiol..

[17] Takashio S., Yamamuro M., Izumiya Y., Sugiyama S., Kojima S., Yamamoto E., Tsujita K., Tanaka T., Tayama S., Kaikita K. (2013). Coronary microvascular dysfunction and diastolic load correlate with cardiac troponin T release measured by a highly sensitive assay in patients with nonischemic heart failure. J. Am. Coll. Cardiol..

[18] Saunders J.T., Nambi V., de Lemos J.A., Chambless L.E., Virani S.S., Boerwinkle E., Hoogeveen R.C., Liu X., Astor B.C., Mosley T.H. (2011). Cardiac troponin T measured by a highly sensitive assay predicts coronary heart disease, heart failure, and mortality in the atherosclerosis risk in communities study. Circulation.

[19] Mortensen S.A., Rosenfeldt F., Kumar A., Dolliner P., Filipiak K.J., Pella D., Alehagen U., Steurer G., Littarru G.P., Q-SYMBIO Study Investigators (2014). The effect of coenzyme Q10 on morbidity and mortality in chronic heart failure: Results from Q-SYMBIO: A randomized double-blind trial. JACC Heart Fail..

[20] Lee T.H., Marcantonio E.R., Mangione C.M., Thomas E.J., Polanczyk C.A., Cook E.F., Sugarbaker D.J., Donaldson M.C., Poss R., Ho K.K. (1999). Derivation and prospective validation of a simple index for prediction of cardiac risk of major noncardiac surgery. Circulation.

[21] Garcia S., Moritz T.E., Goldman S., Littooy F., Pierpont G., Larsen G.C., Reda D.J., Ward H.B., McFalls E.O. (2009). Perioperative complications after vascular surgery are predicted by the revised cardiac risk index but are not reduced in high-risk subsets with preoperative revascularization. Circ. Cardiovasc. Qual. Outcomes.

[22] Devereaux P.J., Sessler D.I., Leslie K., Kurz A., Mrkobrada M., Alonso-Coello P., Villar J.C., Sigamani A., Biccard B.M., Meyhoff C.S. (2014). Clonidine in patients undergoing noncardiac surgery. N. Engl. J. Med..

[23] London M.J., Schwartz G.G., Hur K., Henderson W.G. (2017). Association of perioperative statin use with mortality and morbidity after major noncardiac surgery. JAMA Int. Med..

[24] Smilowitz N.R., Redel-Traub G., Hausvater A., Armanious A., Nicholson J., Puelacher C., Berger J.S. (2019). Myocardial injury after noncardiac surgery: A systematic review and meta-analysis. Cardiol. Rev..

[25] Duceppe E., Heels-Ansdell D., Devereaux P.J. (2020). Preoperative N-terminal pro-B-type natriuretic peptide and cardiovascular Events after noncardiac surgery. Ann. Int. Med..

[26] Turrens J.F. (2003). Mitochondrial formation of reactive oxygen species. J. Physiol..

[27] Lesnefsky E.J., Chen Q., Hoppel C.L. (2016). Mitochondrial metabolism in aging heart. Circ. Res..

[28] Lesnefsky E.J., Chen Q., Tandler B., Hoppel C.L. (2017). Mitochondrial dysfunction and myocardial ischemia-reperfusion: Implications for novel therapies. Annu. Rev. Pharmacol. Toxicol..

[29] Weiss J.N., Korge P., Honda H.M., Ping P. (2003). Role of the mitochondrial permeability transition in myocardial disease. Circ. Res..

[30] Stewart S., Lesnefsky E.J., Chen Q. (2009). Reversible blockade of electron transport with amobarbital at the onset of reperfusion attenuates cardiac injury. Transl. Res..

[31] Halestrap A.P. (2009). What is the mitochondrial permeability transition pore?. J. Mol. Cell. Cardiol..

[32] Halestrap A.P., Clarke S.J., Khaliulin I. (2007). The role of mitochondria in protection of the heart by preconditioning. Biochim. Biophys. Acta.

[33] Chen Q., Paillard M., Gomez L., Li H., Hu Y., Lesnefsky E.J. (2012). Postconditioning modulates ischemia-damaged mitochondria during reperfusion. J. Cardiovasc. Pharmacol..

[34] Chen Q., Paillard M., Gomez L., Ross T., Hu Y., Xu A., Lesnefsky E.J. (2011). Activation of mitochondrial mu-calpain increases AIF cleavage in cardiac mitochondria during ischemia-reperfusion. Biochem. Biophys. Res. Commun..

[35] Green D.R., Reed J.C. (1998). Mitochondria and apoptosis. Science.

[36] Yu S.W., Wang H., Poitras M.F., Coombs C., Bowers W.J., Federoff H.J., Poirier G.G., Dawson T.M., Dawson V.L. (2002). Mediation of poly(ADP-ribose) polymerase-1-dependent cell death by apoptosis-inducing factor. Science.

[37] Maggiorani D., Manzella N., Edmondson D.E., Mattevi A., Parini A., Binda C., Mialet-Perez J. (2017). Monoamine oxidases, oxidative stress, and altered mitochondrial dynamics in cardiac ageing. Oxid. Med. Cell. Longev..

[38] Liang W., Moyzis A.G., Lampert M.A., Diao R.Y., Najor R.H., Gustafsson Å.B. (2020). Aging is associated with a decline in Atg9b-mediated autophagosome formation and appearance of enlarged mitochondria in the heart. Aging Cell.

[39] Dai D.F., Chen T., Wanagat J., Laflamme M., Marcinek D.J., Emond M.J., Ngo C.P., Prolla T.A., Rabinovitch P.S. (2010). Age-dependent cardiomyopathy in mitochondrial mutator mice is attenuated by overexpression of catalase targeted to mitochondria. Aging Cell.

[40] Wu J., Xia S., Kalionis B., Wan W., Sun T. (2014). The role of oxidative stress and inflammation in cardiovascular aging. Biomed. Res. Int..

[41] Sciarretta S., Zhai P., Shao D., Zablocki D., Nagarajan N., Terada L.S., Volpe M., Sadoshima J. (2013). Activation of NADPH oxidase 4 in the endoplasmic reticulum promotes cardiomyocyte autophagy and survival during energy stress through the protein kinase RNA-activated-like endoplasmic reticulum kinase/eukaryotic initiation factor 2alpha/activating transcription factor 4 pathway. Circ. Res..

[42] Chen Q., Allegood J.C., Thompson J., Toldo S., Lesnefsky E.J. (2019). Increased mitochondrial ROS generation from complex III causes mitochondrial damage and increases endoplasmic reticulum stress. FASEB J..

[43] Chen Q., Samidurai A., Thompson J., Hu Y., Das A., Willard B., Lesnefsky E.J. (2020). Endoplasmic reticulum stress-mediated mitochondrial dysfunction in aged hearts. Biochim. Biophys. Acta Mol. Basis Dis..

[44] Zhang Y., Ren J. (2011). Thapsigargin triggers cardiac contractile dysfunction via NADPH oxidase-mediated mitochondrial dysfunction: Role of Akt dephosphorylation. Free Radic. Biol. Med..

[45] Mohsin A.A., Thompson J., Hu Y., Hollander J., Lesnefsky E.J., Chen Q. (2020). Endoplasmic reticulum stress-induced complex I defect: Central role of calcium overload. Arch. Biochem. Biophys..

[46] Chen Q., Thompson J., Hu Y., Das A., Lesnefsky E.J. (2019). Cardiac specific knockout of p53 decreases ER stress-induced mitochondrial damage. Front. Cardiovasc. Med..

[47] Chen Q., Thompson J., Hu Y., Das A., Lesnefsky E.J. (2017). Metformin attenuates ER stress-induced mitochondrial dysfunction. Transl. Res..

[48] Dai D.F., Santana L.F., Vermulst M., Tomazela D.M., Emond M.J., MacCoss M.J., Gollahon K., Martin G.M., Loeb L.A., Ladiges W.C. (2009). Overexpression of catalase targeted to mitochondria attenuates murine cardiac aging. Circulation.

[49] Chen Q., Lesnefsky E.J. (2015). Heart mitochondria and calpain 1: Location, function, and targets. Biochim, Biophys, Acta.

[50] Shintani-Ishida K., Yoshida K. (2015). Mitochondrial m-calpain opens the mitochondrial permeability transition pore in ischemia-reperfusion. Int. J. Cardiol..

[51] Chen M., He H., Zhan S., Krajewski S., Reed J.C., Gottlieb R.A. (2001). Bid is cleaved by calpain to an active fragment in vitro and during myocardial ischemia/reperfusion. J. Biol. Chem..

[52] Chen M., Won D.J., Krajewski S., Gottlieb R.A. (2002). Calpain and mitochondria in ischemia/reperfusion injury. J. Biol. Chem..

[53] Chen Q., Thompson J., Hu Y., Hollander J.M., Lesnefsky E.J. (2018). Activation of mitochondrial calpain 1 leads to degradation of PDH. FASEB J..

[54] Thompson J., Hu Y., Lesnefsky E.J., Chen Q. (2016). Activation of mitochondrial calpain and increased cardiac injury: Beyond AIF release. Am. J. Physiol. Heart Circ. Physiol..

[55] Poncelas M., Inserte J., Aluja D., Hernando V., Vilardosa U., Garcia-Dorado D. (2017). Delayed, oral pharmacological inhibition of calpains attenuates adverse post-infarction remodelling. Cardiovasc. Res..

[56] Singh R.B., Dhalla N.S. (2010). Ischemia-reperfusion-induced changes in sarcolemmal Na+/K+-ATPase are due to the activation of calpain in the heart. Can. J. Physiol. Pharmacol..

[57] Hernando V., Inserte J., Sartorio C.L., Parra V.M., Poncelas-Nozal M., Garcia-Dorado D. (2010). Calpain translocation and activation as pharmacological targets during myocardial ischemia/reperfusion. J. Mol. Cell Cardiol..

[58] Guo A., Hall D., Zhang C., Peng T., Miller J.D., Kutschke W., Grueter C.E., Johnson F.L., Lin R.Z., Song L.-S. (2015). Molecular determinants of calpain-dependent cleavage of junctophilin-2 protein in cardiomyocytes. J. Biol. Chem..

[59] Cao T., Fan S., Zheng D., Wang G., Yu Y., Chen R., Song L., Fan G., Zhang Z., Peng T. (2019). Increased calpain-1 in mitochondria induces dilated heart failure in mice: Role of mitochondrial superoxide anion. Basic Res. Cardiol..

[60] Kumar S., Kain V., Sitasawad S.L. (2012). High glucose-induced Ca2+ overload and oxidative stress contribute to apoptosis of cardiac cells through mitochondrial dependent and independent pathways. Biochim. Biophys. Acta.

[61] Paramo B., Montiel T., Hernandez-Espinosa D.R., Rivera-Martinez M., Moran J., Massieu L. (2013). Calpain activation induced by glucose deprivation is mediated by oxidative stress and contributes to neuronal damage. Int. J. Biochem. Cell Biol..

[62] Thompson J., Maceyka M., Chen Q. (2020). Targeting ER stress and calpain activation to reverse age-dependent mitochondrial damage in the heart. Mech. Ageing Dev..

[63] Dubois-Deruy E., Peugnet V., Turkieh A., Pinet F. (2020). Oxidative stress in cardiovascular diseases. Antioxidants.

[64] Chen Q., Camara A.K., Stowe D.F., Hoppel C.L., Lesnefsky E.J. (2007). Modulation of electron transport protects cardiac mitochondria and decreases myocardial injury during ischemia and reperfusion. Am. J. Physiol. Cell Physiol..

[65] Chen Q., Vazquez E.J., Moghaddas S., Hoppel C.L., Lesnefsky E.J. (2003). Production of reactive oxygen species by mitochondria: Central role of complex III. J. Biol. Chem..

[66] Lesnefsky E.J., Chen Q., Moghaddas S., Hassan M.O., Tandler B., Hoppel C.L. (2004). Blockade of electron transport during Ischemia protects cardiac mitochondria. J. Biol. Chem..

[67] Chen Q., Hoppel C.L., Lesnefsky E.J. (2006). Blockade of electron transport before cardiac ischemia with the reversible inhibitor amobarbital protects rat heart mitochondria. J. Pharmacol. Exp. Ther..

[68] Chen Q., Moghaddas S., Hoppel C.L., Lesnefsky E.J. (2006). Reversible blockade of electron transport during ischemia protects mitochondria and decreases myocardial injury following reperfusion. J. Pharmacol. Exp. Ther..

[69] Aldakkak M., Stowe D.F., Chen Q., Lesnefsky E.J., Camara A.K. (2008). Inhibited mitochondrial respiration by amobarbital during cardiac ischaemia improves redox state and reduces matrix Ca2+ overload and ROS release. Cardiovasc. Res..

[70] Tanaka-Esposito C., Chen Q., Lesnefsky E.J. (2012). Blockade of electron transport before ischemia protects mitochondria and decreases myocardial injury during reperfusion in aged rat hearts. Transl. Res..

[71] Chen Q., Ross T., Hu Y., Lesnefsky E.J. (2012). Blockade of electron transport at the onset of reperfusion decreases cardiac injury in aged hearts by protecting the inner mitochondrial membrane. J. Aging Res..

[72] Han D., Antunes F., Canali R., Rettori D., Cadenas E. (2003). Voltage-dependent anion channels control the release of the superoxide anion from mitochondria to cytosol. J. Biol. Chem..

[73] Sugioka K., Nakano M., Totsune-Nakano H., Minakami H., Tero-Kubota S., Ikegami Y. (1988). Mechanism of O2- generation in reduction and oxidation cycle of ubiquinones in a model of mitochondrial electron transport systems. Biochim. Biophys. Acta.

[74] Mimaki M., Wang X., McKenzie M., Thorburn D.R., Ryan M.T. (2012). Understanding mitochondrial complex I assembly in health and disease. Biochim. Biophys. Acta.

[75] Chen Q., Thompson J., Hu Y., Dean J., Lesnefsky E.J. (2019). Inhibition of the ubiquitous calpains protects complex I activity and enables improved mitophagy in the heart following ischemia-reperfusion. Am. J. Physiol. Cell Physiol..

[76] Galkin A., Brandt U. (2005). Superoxide radical formation by pure complex I (NADH:ubiquinone oxidoreductase) from Yarrowia lipolytica. J. Biol. Chem..

[77] Bazil J.N., Pannala V.R., Dash R.K., Beard D.A. (2014). Determining the origins of superoxide and hydrogen peroxide in the mammalian NADH:ubiquinone oxidoreductase. Free Radic. Biol. Med..

[78] Brand M.D., Esteves T.C. (2005). Physiological functions of the mitochondrial uncoupling proteins UCP2 and UCP3. Cell Metab..

[79] Ross T., Szczepanek K., Bowler E., Hu Y., Larner A., Lesnefsky E.J., Chen Q. (2013). Reverse electron flow-mediated ROS generation in ischemia-damaged mitochondria: Role of complex I inhibition vs. depolarization of inner mitochondrial membrane. Biochim. Biophys. Acta.

[80] Kushnareva Y., Murphy A.N., Andreyev A. (2002). Complex I-mediated reactive oxygen species generation: Modulation by cytochrome c and NAD(P)+ oxidation-reduction state. Biochem. J..

[81] Grivennikova V.G., Vinogradov A.D. (2006). Generation of superoxide by the mitochondrial Complex, I. Biochim. Biophys. Acta.

[82] Chen Q., Lesnefsky E.J. (2008). Ischemic damage to the mitochondrial electron transport chain favors opening of the permeability transition pore. FASEB J..

[83] Chen Q., Lesnefsky E.J. (2006). Depletion of cardiolipin and cytochrome c during ischemia increases hydrogen peroxide production from the electron transport chain. Free Radic. Biol. Med..

[84] Gomez L.A., Monette J.S., Chavez J.D., Maier C.S., Hagen T.M. (2009). Supercomplexes of the mitochondrial electron transport chain decline in the aging rat heart. Arch. Biochem. Biophys..

[85] Rosca M., Minkler P., Hoppel C.L. (2011). Cardiac mitochondria in heart failure: Normal cardiolipin profile and increased threonine phosphorylation of complex IV. Biochim. Biophys. Acta.

[86] Genova M.L., Lenaz G. (2014). Functional role of mitochondrial respiratory supercomplexes. Biochim. Biophys. Acta.

[87] Maranzana E., Barbero G., Falasca A.I., Lenaz G., Genova M.L. (2013). Mitochondrial respiratory supercomplex association limits production of reactive oxygen species from complex I. Antioxid. Redox. Sign..

[88] Jang S., Javadov S. (2018). Elucidating the contribution of ETC complexes I and II to the respirasome formation in cardiac mitochondria. Sci. Rep..

[89] Chen Q., Moghaddas S., Hoppel C.L., Lesnefsky E.J. (2008). Ischemic defects in the electron transport chain increase the production of reactive oxygen species from isolated rat heart mitochondria. Am. J. Physiol. Cell. Physiol..

[90] Chen Q., Thompson J., Hu Y., Lesnefsky E.J. (2020). Cardiomyocyte specific deletion of p53 decreases cell injury during ischemia-reperfusion: Role of Mitochondria. Free Radic. Biol. Med..

[91] Chouchani E.T., Pell V.R., Gaude E., Aksentijevic D., Sundier S.Y., Robb E.L., Logan A., Nadtochiy S.M., Ord E.N.J., Smith A.C. (2014). Ischaemic accumulation of succinate controls reperfusion injury through mitochondrial ROS. Nature.

[92] Pell V.R., Chouchani E.T., Murphy M.P., Brookes P.S., Krieg T. (2016). Moving forwards by blocking back-flow: The yin and yang of mi therapy. Circ. Res..

[93] Hadrava Vanova K., Kraus M., Neuzil J., Rohlena J. (2020). Mitochondrial complex II and reactive oxygen species in disease and therapy. Redox. Rep..

[94] Markevich N.I., Markevich L.N., Hoek J.B. (2020). Computational modeling analysis of generation of reactive oxygen species by mitochondrial assembled and disintegrated complex II. Front. Physiol..

[95] Gille L., Nohl H. (2001). The ubiquinol/bc1 redox couple regulates mitochondrial oxygen radical formation. Arch. Biochem. Biophys..

[96] St-Pierre J., Buckingham J.A., Roebuck S.J., Brand M.D. (2002). Topology of superoxide production from different sites in the mitochondrial electron transport chain. J. Biol. Chem..

[97] Moghaddas S., Hoppel C.L., Lesnefsky E.J. (2003). Aging defect at the Qo site of complex III augments oxyradical production in rat heart interfibrillar mitochondria. Arch. Biochem. Biophys..

[98] Kaludercic N., Mialet-Perez J., Paolocci N., Parini A., Di Lisa F. (2014). Monoamine oxidases as sources of oxidants in the heart. J. Mol. Cell Cardiol..

[99] Kaludercic N., Takimoto E., Nagayama T., Feng N., Lai E.W., Bedja D., Chen K., Gabrielson K.L., Blakely R.D., Shih J.C. (2010). Monoamine oxidase A-mediated enhanced catabolism of norepinephrine contributes to adverse remodeling and pump failure in hearts with pressure overload. Circ. Res..

[100] Menazza S., Blaauw B., Tiepolo T., Toniolo L., Braghetta P., Spolaore B., Reggiani C., di Lisa F., Bonaldo P., Canton M. (2010). Oxidative stress by monoamine oxidases is causally involved in myofiber damage in muscular dystrophy. Hum. Mol. Genet..

[101] Mir H.A., Ali R., Mushtaq U., Khanday F.A. (2020). Structure-functional implications of longevity protein p66Shc in health and disease. Ageing Res. Rev..

[102] Chen Q., Yin G., Stewart S., Hu Y., Lesnefsky E.J. (2010). Isolating the segment of the mitochondrial electron transport chain responsible for mitochondrial damage during cardiac ischemia. Biochem. Biophys. Res. Commun..

[103] Migliaccio E., Giorgio M., Pelicci P.G. (2013). p53 and aging: Role of p66Shc. Aging.

[104] Hao C., Wu X., Zhou R., Zhang H., Zhou Y., Wang X., Feng Y., Mei L., He C., Cai X. (2019). Downregulation of p66Shc can reduce oxidative stress and apoptosis in oxidative stress model of marginal cells of stria vascularis in Sprague dawley rats. Drug Des. Devel. Ther..

[105] Sharifi-Rad M., Anil Kumar N.V., Zucca P., Varoni E.M., Dini L., Panzarini E., Rajkovic J., Valere P., Fokou T., Azzini E. (2020). Lifestyle, oxidative stress, and antioxidants: Back and forth in the pathophysiology of chronic diseases. Front. Physiol..

[106] Hu W., Wang H., Shu Q., Chen M., Xie L. (2020). Green tea polyphenols modulated cerebral SOD expression and endoplasmic reticulum stress in cardiac arrest/cardiopulmonary resuscitation rats. Biomed. Res. Int..

[107] Strassburger M., Bloch W., Sulyok S., Schüller J., Keist A.F., Schmidt A., Wenk J., Peters T., Wlaschek M., Lenart J. (2005). Heterozygous deficiency of manganese superoxide dismutase results in severe lipid peroxidation and spontaneous apoptosis in murine myocardium in vivo. Free Radic. Biol. Med..

[108] Sepasi Tehrani H., Moosavi-Movahedi A.A. (2018). Catalase and its mysteries. Prog. Biophys. Mol. Biol..

[109] Aluri H.S., Simpson D.C., Allegood J.C., Hu Y., Szczepanek K., Gronert S., Chen Q., Lesnefsky E.J. (2014). Electron flow into cytochrome c coupled with reactive oxygen species from the electron transport chain converts cytochrome c to a cardiolipin peroxidase: Role during ischemia-reperfusion. Biochim. Biophys. Acta.

[110] Detienne G., De Haes W., Mergan L., Edwards S.L., Temmerman L., Van Bael S. (2018). Beyond ROS clearance: Peroxiredoxins in stress signaling and aging. Ageing Res. Rev..

[111] Poynton R.A., Hampton M.B. (2014). Peroxiredoxins as biomarkers of oxidative stress. Biochim. Biophys. Acta.

[112] Kumar V., Kitaeff N., Hampton M.B., Cannell M.B., Winterbourn C.C. (2009). Reversible oxidation of mitochondrial peroxiredoxin 3 in mouse heart subjected to ischemia and reperfusion. FEBS Lett..

[113] Matsushima S., Ide T., Yamato M., Matsusaka H., Hattori F., Ikeuchi M., Kubota T., Sunagawa K., Hasegawa Y., Kurihara T. (2006). Overexpression of mitochondrial peroxiredoxin-3 prevents left ventricular remodeling and failure after myocardial infarction in mice. Circulation.

[114] Eismann T., Huber N., Shin T., Kuboki S., Galloway E., Wyder M., Edwards M.J., Greis K.D., Shertzer H.G., Fisher A.B. (2009). Peroxiredoxin-6 protects against mitochondrial dysfunction and liver injury during ischemia-reperfusion in mice. Am. J. Physiol. Gastrointest. Liver Physiol..

[115] Karaduleva E.V., Mubarakshina E.K., Sharapov M.G., Volkova A.E., Pimenov O.Y., Ravin V.K., Kokoz Y.M., Novoselov V.I. (2016). Cardioprotective effect of modified peroxiredoxins in retrograde perfusion of isolated rat heart under conditions of oxidative stress. Bull. Exp. Biol. Med..

[116] Borchert A., Wang C.C., Ufer C., Schiebel H., Savaskan N.E., Kuhn H. (2006). The role of phospholipid hydroperoxide glutathione peroxidase isoforms in murine embryogenesis. J. Biol. Chem..

[117] Park T.J., Park J.H., Lee G.S., Lee J.Y., Shin J.H., Kim M.W., Kim Y.S., Kim J.Y., Oh K.-J., Han B.-S. (2019). Quantitative proteomic analyses reveal that GPX4 downregulation during myocardial infarction contributes to ferroptosis in cardiomyocytes. Cell Death. Dis..

[118] Ungvari Z., Orosz Z., Rivera A., Labinskyy N., Xiangmin Z., Olson S., Podlutsky A., Csiszar A. (2007). Resveratrol increases vascular oxidative stress resistance. Am. J. Physiol. Heart Circ. Physiol..

[119] Bajpai V.K., Alam M.B., Quan K.T., Kwon K.R., Ju M.K., Choi H.J., Lee J.S., Yoon J.-I., Majumder R., Rather I.A. (2017). Antioxidant efficacy and the upregulation of Nrf2-mediated HO-1 expression by (+)-lariciresinol, a lignan isolated from Rubia philippinensis, through the activation of p38. Sci. Rep..

[120] Clerk A. (2003). The radical balance between life and death. J. Mol. Cell Cardiol..

[121] Tsushima M., Lium J., Hirao W., Yamazaki H., Tomita H., Itoh K. (2020). Emerging evidence for crosstalk between Nrf2 and mitochondria in physiological homeostasis and in heart disease. Arch. Pharm. Res..

[122] Carlström K.E., Ewing E., Granqvist M., Gyllenberg A., Aeinehband S., Enoksson S.L., Checa A., Badam T.V.S., Huang J., Gomez-Cabrero D. (2019). Therapeutic efficacy of dimethyl fumarate in relapsing-remitting multiple sclerosis associates with ROS pathway in monocytes. Nat. Commun..

[123] Inoue Y., Uchiyama A., Sekiguchi A., Yamazaki S., Fujiwara C., Yokoyama Y., Ogino S., Torii R., Hosoi M., Akai R. (2020). Protective effect of dimethyl fumarate for the development of pressure ulcers after cutaneous ischemia-reperfusion injury. Wound Rep. Regen..

[124] Ibrahim S.G., El-Emam S.Z., Mohamed E.A., Abd Ellah M.F. (2020). Dimethyl fumarate and curcumin attenuate hepatic ischemia/reperfusion injury via Nrf2/HO-1 activation and anti-inflammatory properties. Int. Immunopharmacol..

[125] Ernster L., Dallner G. (1995). Biochemical, physiological and medical aspects of ubiquinone function. Biochim. Biophys. Acta.

[126] Bhagavan H.N., Chopra R.K. (2006). Coenzyme Q10: Absorption, tissue uptake, metabolism and pharmacokinetics. Free Radic. Res..

[127] Papucci L., Schiavone N., Witort E., Donnini M., Lapucci A., Tempestini A., Formigli L., Zecchi-Orlandini S., Orlandini G., Carella G. (2003). Coenzyme q10 prevents apoptosis by inhibiting mitochondrial depolarization independently of its free radical scavenging property. J. Biol. Chem..

[128] Nutrition & Metabolism. https://nutritionandmaetabolism.biomedcentral.com/.

[129] Choi H., Park H.H., Koh S.H., Choi N.Y., Yu H.J., Park J., Lee Y.J., Lee K.-Y. (2012). Coenzyme Q10 protects against amyloid beta-induced neuronal cell death by inhibiting oxidative stress and activating the P13K pathway. Neurotoxicology.

[130] Maulik N., Yoshida T., Engelman R.M., Bagchi D., Otani H., Das D.K. (2000). Dietary coenzyme Q(10) supplement renders swine hearts resistant to ischemia-reperfusion injury. Am. J. Physiol. Heart Circ. Physiol..

[131] Rosenfeldt F., Marasco S., Lyon W., Wowk M., Sheeran F., Bailey M., Esmore D., Davis B., Pick A., Rabinov M. (2005). Coenzyme Q10 therapy before cardiac surgery improves mitochondrial function and in vitro contractility of myocardial tissue. J. Thorac. Cardiovasc. Surg..

[132] Leong J.Y., van der Merwe J., Pepe S., Bailey M., Perkins A., Lymbury R., Esmore D., Marasco S., Rosenfeldt F. (2010). Perioperative metabolic therapy improves redox status and outcomes in cardiac surgery patients: A randomised trial. Heart Lung. Circ..

[133] Anderson R., Prolla T. (2009). PGC-1alpha in aging and anti-aging interventions. Biochim. Biophys. Acta.

[134] Wagner A.E., Ernst I.M., Birringer M., Sancak O., Barella L., Rimbach G. (2012). A combination of lipoic acid plus coenzyme Q10 induces PGC1α, a master switch of energy metabolism, improves stress response, and increases cellular glutathione levels in cultured C2C12 skeletal muscle cells. Oxid. Med. Cell Longev..

[135] Nagib M.M., Tadros M.G., Al-Khalek H.A.A., Rahmo R.M., Sabri N.A., Khalifa A.E., Masoud S.I. (2018). Molecular mechanisms of neuroprotective effect of adjuvant therapy with phenytoin in pentylenetetrazole-induced seizures: Impact on Sirt1/NRF2 signaling pathways. Neurotoxicology.

[136] Tian G., Sawashita J., Kubo H., Nishio S.Y., Hashimoto S., Suzuki N., Yoshimura H., Tsuruoka M., Wang Y., Liu Y. (2014). Ubiquinol-10 supplementation activates mitochondria functions to decelerate senescence in senescence-accelerated mice. Antioxid. Redox. Sign..

[137] Hocum Stone L., Chappuis E., Wright C., Kelly R.F., McFalls E.O. (2019). CoQ(10) enhances PGC1α and increases expression of mitochondrial antioxidant proteins in chronically ischemic swine myocardium. Nutr. Metab..

[138] Mugoni V., Postel R., Catanzaro V., De Luca E., Turco E., Digilio G., Silengo S., Murphy M.P., Medana C., Stainier D.Y.R. (2013). Ubiad1 is an antioxidant enzyme that regulates eNOS activity by CoQ_10_ synthesis. Cell.

[139] Mazidi M., Kengne A.P., Banach M. (2018). Effects of coenzyme Q10 supplementation on plasma C-reactive protein concentrations: A systematic review and meta-analysis of randomized controlled trials. Pharmacol. Res..

[140] Heinrich P.C., Castell J.V., Andus T. (1990). Interleukin-6 and the acute phase response. Biochem. J..

[141] Fan L., Feng Y., Chen G.C., Qin L.Q., Fu C.L., Chen L.H. (2017). Effects of coenzyme Q10 supplementation on inflammatory markers: A systematic review and meta-analysis of randomized controlled trials. Pharmacol. Res..

[142] Schmelzer C., Lorenz G., Rimbach G., Döring F. (2009). In vitro effects of the reduced form of coenzyme Q(10) on secretion levels of TNF-alpha and chemokines in response to LPS in the human monocytic cell line THP-1. J Clin. Biochem. Nutr..

[143] Ebadi M., Sharma S.K., Wanpen S., Amornpan A. (2004). Coenzyme Q10 inhibits mitochondrial complex-1 down-regulation and nuclear factor-kappa B activation. J. Cell Mol. Med..

[144] Hernández-Camacho J.D., Bernier M., López-Lluch G., Navas P. (2018). Coenzyme Q(10) supplementation in aging and disease. Front. Physiol..

[145] Del Pozo-Cruz J., Rodríguez-Bies E., Navas-Enamorado I., Del Pozo-Cruz B., Navas P., López-Lluch G. (2014). Relationship between functional capacity and body mass index with plasma coenzyme Q10 and oxidative damage in community-dwelling elderly-people. Exp. Gerontol..

[146] McGarry A., McDermott M., Kieburtz K., de Blieck E.A., Beal F., Marder K., Ross C., Shoulson I., Gilbert P., Mallonee W.M. (2017). A randomized, double-blind, placebo-controlled trial of coenzyme Q10 in Huntington disease. Neurology.

[147] González-Guardia L., Yubero-Serrano E.M., Delgado-Lista J., Perez-Martinez P., Garcia-Rios A., Marin C., Camargo A., Delgado-Casado N., Roche H.M., Perez-Jimenez F. (2015). Effects of the Mediterranean diet supplemented with coenzyme q10 on metabolomic profiles in elderly men and women. J. Gerontol. A. Biol. Sci. Med. Sci..

[148] Lee B.J., Huang Y.C., Chen S.J., Lin P.T. (2012). Coenzyme Q10 supplementation reduces oxidative stress and increases antioxidant enzyme activity in patients with coronary artery disease. Nutrition.

[149] Makhija N., Sendasgupta C., Kiran U., Lakshmy R., Hote M.P., Choudhary S.K., Airan B., Abrahame R. (2008). The role of oral coenzyme Q10 in patients undergoing coronary artery bypass graft surgery. J. Cardiothorac. Vasc. Anesth..

[150] Alehagen U., Aaseth J., Johansson P. (2015). Reduced cardiovascular mortality 10 years after supplementation with selenium and coenzyme Q10 for four years: Follow-up results of a prospective randomized double-blind placebo-controlled trial in elderly citizens. PLoS ONE.

[151] Rivara M.B., Yeung C.K., Robinson-Cohen C., Phillips B.R., Ruzinski J., Rock D., Linke L., Shen D.D., Ikizler T.A., Himmelfarb J. (2017). Effect of coenzyme Q(10) on biomarkers of oxidative stress and cardiac function in hemodialysis patients: The CoQ(10) biomarker trial. Am. J. Kidney Dis..

[152] Onur S., Niklowitz P., Jacobs G., Lieb W., Menke T., Döring F. (2015). Association between serum level of ubiquinol and NT-proBNP, a marker for chronic heart failure, in healthy elderly subjects. Biofactors.

[153] Alehagen U., Aaseth J., Alexander J., Johansson P. (2018). Still reduced cardiovascular mortality 12 years after supplementation with selenium and coenzyme Q10 for four years: A validation of previous 10-year follow-up results of a prospective randomized double-blind placebo-controlled trial in elderly. PLoS ONE.

[154] Lee B.J., Tseng Y.F., Yen C.H., Lin P.T. (2013). Effects of coenzyme Q10 supplementation (300 mg/day) on antioxidation and anti-inflammation in coronary artery disease patients during statins therapy: A randomized, placebo-controlled trial. Nutr. J..

[155] Zhao L., Cheng G., Jin R., Afzal M.R., Samanta A., Xuan Y.T., Girgis M., Elias H.K., Zhu Y., Davani A. (2016). Deletion of interleukin-6 attenuates pressure overload-induced left ventricular hypertrophy and dysfunction. Circ. Res..

[156] Bradham W.S., Ormseth M.J., Oeser A., Solus J.F., Gebretsadik T., Shintani A., Stein C.M. (2014). Insulin resistance is associated with increased concentrations of NT-proBNP in rheumatoid arthritis: IL-6 as a potential mediator. Inflammation.

[157] Jarai R., Kaun C., Weiss T.W., Speidl W.S., Rychli K., Maurer G., Huber K., Wojta J. (2009). Human cardiac fibroblasts express B-type natriuretic peptide: Fluvastatin ameliorates its up-regulation by interleukin-1alpha, tumour necrosis factor-alpha and transforming growth factor-beta. J. Cell Mol. Med..

[158] de Frutos F., Gea A., Hernandez-Estefania R., Rabago G. (2015). Prophylactic treatment with coenzyme Q10 in patients undergoing cardiac surgery: Could an antioxidant reduce complications? A systematic review and meta-analysis. Interact. Cardiovasc. Thorac. Surg..

[159] Di Lorenzo A., Iannuzzo G., Parlato A., Cuomo G., Testa C., Coppola M., D’Ambrosio G., Oliviero D.A., Sarullo S., Vitale G. (2020). Clinical evidence for Q10 coenzyme supplementation in heart failure: From energetics to functional improvement. J. Clin. Med..

[160] Orlando P., Sabbatinelli J., Silvestri S., Marcheggiani F., Cirilli I., Dludla P.V., Molardi A., Nicolini F., Tiano L. (2020). Ubiquinol supplementation in elderly patients undergoing aortic valve replacement: Biochemical and clinical aspects. Aging.

[161] Taggart D.P., Jenkins M., Hooper J., Hadjinikolas L., Kemp M., Hue D., Bennett G. (1996). Effects of short-term supplementation with coenzyme Q10 on myocardial protection during cardiac operations. Ann. Thorac. Surg..

[162] Judy W.V., Stogsdill W.W., Folkers K. (1993). Myocardial preservation by therapy with coenzyme Q10 during heart surgery. Clin. Investig.

